# Genetic association of *HCRTR2, ADH4* and *CLOCK* genes with cluster headache: a Chinese population-based case-control study

**DOI:** 10.1186/s10194-017-0831-1

**Published:** 2018-01-09

**Authors:** Zhiliang Fan, Lei Hou, Dongjun Wan, Ran Ao, Dengfa Zhao, Shengyuan Yu

**Affiliations:** 1 Department of Neurology, Chinese People’s Liberation Army General Hospital, Fuxing Road 28, Haidian District, Beijing, 100853 China; 2 The third department of Neurology, Affiliated Xingtai People’s Hospital of Hebei Medical University, Xingtai, Hebei Province 054000 China

**Keywords:** Cluster headache, Gene polymorphism, *HCRTR2*, *ADH4*, *Clock*

## Abstract

**Background:**

Cluster headache (CH), a rare primary headache disorder, is currently thought to be a genetic susceptibility which play a role in CH susceptibility. A large numbers of genetic association studies have confirmed that the *HCRTR2* (Hypocretin Receptor 2) SNP rs2653349, and the *ADH4* (Alcohol Dehydrogenase 4) SNP rs1126671 and rs1800759 polymorphisms are linked to CH. In addition, the *CLOCK* (Circadian Locomotor Output Cycles Kaput) gene is becoming a research hotspot for CH due to encoding a transcription factor that serves as a basic driving force for circadian rhythm in humans. The purpose of this study was to evaluate the association between CH and the *HCRTR2, ADH4* and *CLOCK* genes in a Chinese CH case–control sample.

**Methods:**

We genotyped polymorphisms of nine single nucleotide polymorphisms (SNPs) in the *HCRTR2, ADH4* and *CLOCK* genes to perform an association study on a Chinese Han CH case-control sample (112 patients and 192 controls),using Sequenom MALDI-TOF mass spectrometry iPLEX platform. The frequencies and distributions of genotypes and haplotypes were statistically compared between the case and control groups to identify associations with CH. The effects of SNPs on CH were further investigated by multiple logistic regression.

**Results:**

The frequency of the *HCRTR2* SNP rs3800539 GA genotype was significantly higher in cases than in controls (48.2% vs.37.0%). The GA genotypes was associated with a higher CH risk (OR = 1.483, 95% CI: 0.564-3.387, *p* = 0.038), however, after Bonferroni correction, the association lost statistical significance. Haplotype analysis of the *HCRTR2* SNPs showed that among eight haplotypes, only H1-GTGGGG was linked to a reduced CH risk (44.7% vs. 53.1%, OR = 0.689, 95% CI =0.491~0.966, *p* = 0.030). No significant association of *ADH4*, *CLOCK* SNPs with CH was statistically detected in the present study.

**Conclusions:**

Association between *HCRTR2, ADH4,CLOCK* gene polymorphisms and CH was not significant in the present study, however, haplotype analysis indicated H1-GTGGGG was linked to a reduced CH risk.

**Electronic supplementary material:**

The online version of this article (10.1186/s10194-017-0831-1) contains supplementary material, which is available to authorized users.

## Background

Cluster headache (CH) is a relatively rare, severe type of primary trigeminal autonomic cephalalgia [1]. It has clinically unique features of excruciating unilateral periorbital pain with recurrent short-lasting attacks (15 to 180 min), accompanied by cranial autonomic symptoms, such as nasal congestion, runny nose (rhinorrhea), tears and eye congestion. According to the cluster period or the duration of remission, CH can be divided into episodic and chronic [2].

The etiology and pathogenic mechanism of cluster headache has been not clarified absolutely, however, recent research has shown that genetic and environmental factors have potential relations with CH [3]. Currently, about 5%-20% of CH patients had CH family history. Familial occurrence of CH has been presented at least four studies, compared with the general population, the risk of direct lineal descendants increased by 14 to 39 times, while the risk of branch lineal descendants increased by 2 to 8 times [4–7]. CH in monozygotic twins were also observed to be suffered from paroxysmal tachycardia [8–10]. Russell et al. (1995) revealed that autosomal dominant genes play a significant role in CH in some families [11]. Therefore, CH is believed to be a genetic susceptibility disease, but at present, the type and the number of genes involved are still unclear.

Previous study hypothesized that the pathogenesis of CH is associated with the changes of circadian rhythm and neuroendocrine disorders in the hypothalamus [12]. The hypothalamus gastrin-releasing peptides (hypocretin, HCRTs) are neuropeptides, which are synthesized by neurons located in the hypothalamus lateral area to regulate arousal, wakefulness and appetite. It is observed that there is a connection between these peptides and nociceptive phenomena in CH. A possible explanation for this mechanism is that HCRTS binds to G-protein coupled receptors (GPCRs), including *HCRTR*1 and *HCRTR*2, to regulate various physiological responses such as sleep awakening, dietary energy metabolism and blood pressure [13, 14]. Four studies revealed significant association of *HCTR* gene polymorphism with CH. A significant association between a 1246G-A polymorphism (rs2653349) in the *HCRTR2* gene and CH has been independently reported by two research groups [15–18].

About 52%–79% of CH occur during the onset of the disease while alcohol can trigger an episode during a cluster period [19]. Alcohol is metabolizatied by alcohol dehydrogenase (ADH), which is a group of dehydrogenase enzymes mainly distributed in the liver and gastrointestinal tract [20]. Ethanol dehydrogenase 4 (*ADH4*), encoded by the *ADH4* gene, is a member of the ADH family, and associated with many diseases including Parkinson’s disease, alcohol and drug-dependent syndromes [21, 22]. Alcohol is a well-known trigger factor for CH attacks during the active phases of the disease. Three studies have been made,two Italian groups revealed significant association of the rs1126671 locus in the *ADH4* gene with CH incidence,one of the experiments have also found the association between the *ADH4* SNP rs1800759 and CH,but those results were not confirmed in a large Swedish case–control cohort study [23–25].

CH is characterized by periodic series of episodes. Therefore, the abnormal internal CLOCK function of hypothalamus is hypothesized to associate with CH pathophysiology. *CLOCK* (Circadian Locomotor Output Cycles Kaput) gene, as a candidate gene for CH, which encodes a protein belonging to the superfamily of the basic helix-loop-helix-PAS transcription factor, affects the persistence and the period of circadian rhythm in humans [26]. Recently,four studies have been conducted to investigate the association of the polymorphism of the human *CLOCK* gene (rs1801260) with CH,however, no consistent evidence for association of *CLOCK* with CH was observed yet [24, 27–29].

The identification of association of genes with CH seems to be a difficult task. To date, there is a limited number of the research on genetic association of polymorphisms in the *HCRTR*2, *ADH4* and *CLOCK* genes with CH with contradictory results. Knowledge and understanding of genes for CH derive primarily from studies in Western populations. The molecular association profile of CH in Asia, particularly in Chinese patients have not been fully studied. The purpose of this study was to evaluate the association between CH and the *HCRTR*2 (rs10498801, rs2653342,rs2653349, rs3122156, rs3800539, rs9357855), *ADH4*(rs1126671、rs1800759) and *CLOCK* (rs1801260) genes, estimating the frequency of different gene haplotypes in a Chinese case–control cohort population. Our study provided abundant information and reference value for genes involved with CH in Chinese Han population.

## Methods

### Study samples

A total of 112 CH patients (100 men and 12 women) were recruited by neurologists in the International Headache Center of the Department of Neurology of the Chinese PLA General Hospital between September 2014 and October 2017. The mean age of the patients is 35.43 years, with a standard deviation of 10.56 years. The CH was diagnosed according to the International Classification of Headache Disorders (ICHD-III beta). The candidates in the control group were matched with the candidates in the case group by age and gender, and recruited from physical examination center of the same geographic areas, consisting of 192 unrelated non-headache healthy volunteers (170 men and 22 women). The mean age of the healthy volunteers is 35.81 years, with a standard deviation of 10.87 years. All candidates (CH and controls) with stroke, tumors or a history of mental disease were excluded. Both CH patients and controls were from the Chinese Han population according to the selection criteria as follows: 1) registered as the ethic Han, 2) their parents were registered as the ethnic Han, and 3) the families have settled in China for more than 5 generations without marrying other ethics and intermarrying with other nationalities. The clinical profiles and characteristics of both CH patients and controls in the study is showed in Table 1.

**Table 1:** Clinical characteristics of the study population

	CH (*n* = 112)	Control (*n* = 192)	*P* value
Age (mean ± SD)	35.43 ± 10.56	35.81 ± 10.87	0.398
Sex (M/F)	100/12	170/22	0.959
Age at onset (years)	25.53 ± 9.28		
Headache duration			
< 1 h	43		
1 h-2 h	43		
2 h-3 h	26		
Type of attack			
Episodic type	99		
Chronic type	13		
Drinking induced			
yes	22		
no	90		
Family history of Cluster headache			
yes	9		
no	103		

### DNA isolation and genotyping

A 200 mL of peripheral blood was collected from each of patients and controls. Genomic DNA was extracted from the lymphocytes present in 200 mL peripheral blood using the TIANamp Blood DNA Kit (Tian-gen Biotech, Beijing, China), according to standard manufacturer’s protocols. The primers are designed by Agena Bioscience ADS2.0 software (http://agenabio.com/), and synthesized by Beijing LiuheHuada Gene Technology Co., Ltd. (Beijing, China). The sequences of the primers are listed in the Additional file 1: Table S1.

The multiple PCR genotyping is conducted in GeneAmp ® PCR System 9700 Dual 384-Well Sample Block Module according to the manufacturer’s instruction (Agena Bioscience, USA). PCR products were purified to remove salts by cation-exchange resin. The chip was prepared by using microarray Nanodispenser (MassARRAY Nanodispenser) on the SpectroCHIP microarray (SpectroCHIP). Single nucleotide polymorphism (SNP) genotyping was performed by using MassARRAY Analyzer 4 System (http://agenabio.com/products/massarray-system/). The quality and classification of SNP data were evaluated by using RT MassARRAY™ software. SpectroREAD software (Squenom, Inc. San Diego, USA) was used for automatically data collection and processing, subsequently, analysis and load into database by comparing with the theoretical value. Typer 4.0 software (Sequenom, Inc. San Diego, USA) (NASDAQ: SQNM) was used to analyze the original testing data of the test project to normalize the testing results.

### Statistical analysis

The basic characteristics of the case and control groups were measured by t-test or chi-square test. The Hardy-Weinberg equilibrium was verified for all subjects. The distribution of SNPs (genotypes and alleles) of the three genes was compared with the chi-square test, and the odds ratios (OR) with 95% confidence intervals (CI) was estimated. The significant level was corrected during multiple comparisons by the Bonferroni correction, and a *p* value <0.05/number of comparisons was considered statistically significant. SHEsis was used to construct the single body of 6 SNPs in the *HCRTR*2 gene and compare the distribution differences between the control and the case groups. Multivariate logistic regression was used to analyze the effects of SNPs and related factors on CH for detection of the ultimate risk factors and calculation of the OR and 95% CI. *P* < 0.05 was considered significant.

## Results

### Clinical characteristics of the case-control study population

The study population consisted of 112 patients (100 males and 12 females) and 192 controls (170 males and 22 females). The mean age of the cases was 35.43 ± 10.56 years, while the mean age of the 192 controls was 35.81 ± 10.87 years. T-test showed no significant differences in age and gender between the case and control groups (*p* = 0.398/ *p* = 0.959, respectively) (Table 1).

### Genotype and allele frequency of the polymorphisms between the cases and controls genotype and allelic analysis

All the genes showed the polymorphisms of SNPs except for rs1126671 that was excluded from the statistical analysis. The allele frequency at each locus and the genotype distributions of all SNPs were in the Hardy–Weinberg equilibrium in both patients and controls (*P* > 0.05). The genotype and allele frequencies of all 8 SNPs in cases and controls are shown in Table 2. The frequency of the rs3800539 GA genotypes was significantly higher in cases than in controls (48.2% vs.37.0%). The GA genotypes was associated with a higher CH risk (OR = 1.483, 95% CI: 0.564-3.387, *p* = 0.038), However, there was no signifcant association after the correction for multiple tests (*p*^corr^ = 0.114). There was no significant difference in the distributions of genotypes and alleles of other SNPs between patients and controls (Table 2).

**Table 2:** Genotype and allele frequency of the polymorphisms between cases and controls

SNP		CH (n = 112)	Controls (n = 192)	*p*-value	*p* ^corr^	Crude OR (95% CI)	Adjusted OR ^a^(95% CI)
*HCRTR2*							
rs10498801(G > A)	GG	53(0.473)	95(0.495)			1	1
	GA	46(0.411)	83(0.432)	0.979	1	1.003(0.798-1.261)	1.014(0.596-1.631)
	AA	13(0.116)	14(0.073)	0.224	0.672	1.664(0.728-3.803)	1.634(0.863-4.125)
HWE *p*-value		0.536	0.473				
Allele	G	152(0.679)	273(0.711)			1	–
	A	72(0.321)	111(0.289)	0.599		1.110(0.771-1.568)	–
rs2653342(A > G)	GG	100(0.893)	175(0.911)			1	1
	GA	11(0.098)	17(0.089)	0.760	1	1.132(0.510-2.513)	1.141(0.519-2.404)
	AA	1(0.009)	0(0.000)	0.281	0.843	3.485(0.312-38.91)	–
HWE *p*-value		0.281	0.521				
Allele	G	211(0.942)	367(0.956)			1	–
	A	13(0.058)	17(0.044)	0.454		1.330(0.634-2.793)	–
rs2653349(G > A)	GG	98(0.875)	176(0.917)			1	1
	GA	13(0.116)	16(0.083)	0.336	1	1.459(0.674-3.159)	1.536(0.709-3.331)
	AA	1(0.009)	0(0.000)	0.268	0.804	0.989(0.969-1.010)	–
HWE *p*-value		0.452	0.546				
Allele	G	209(0.933)	368(0.958)			1	–
	A	15(0.067)	16(0.042)	0.171		1.651(0.800-3.407)	–
rs3122156(T > G)	TT	56(0.500)	106(0.552)			1	1
	GT	47(0.420)	74(0.385)	0.460	1	1.202(0.738-1.959)	1.127(0.427-2.974)
	GG	9(0.080)	12(0.063)	0.455	1	1.420(0.564-3.573)	1.314(0.507-3.410)
HWE *p*-value		0.843	0.847				
Allele	G	159(0.710)	286(0.745)			1	–
	T	65(0.290)	98(0.255)	0.348		1.193(0.825-1.725)	–
rs3800539(G > A)	GG	48(0.429)	106(0.552)			1	1
	GA	54(0.482)	71(0.370)	0.038	0.114	1.680(1.027-2.746)	1.483(0.564-3.387)
	AA	10(0.089)	15(0.078)	0.382	1	1.472(0.617-3.513)	0.835(0.339-2.057)
HWE *p*-value		0.342	0.522				
Allele	G	150(0.670)	283(0.737)			1	–
	A	74(0.330)	101(0.263)	0.707		1.382(0.965-1.980)	–
rs9357855(G > A)	GG	65(0.580)	124(0.646)			1	1
	GA	41(0.366)	61(0.318)	0.326	0.978	1.282(0.780-2.107)	1.423(0.444-4.565)
	AA	6(0.054)	7(0.036)	0.390	1	1.635(0.528-5.067)	1.694(0.546-5.257)
HWE *p*-value		0.887	0.881				
Allele	G	171(0.763)	309(0.805)			1	–
	A	53(0.237)	75(0.195)	0.228		1.277(0.857-1.902)	–
*ADH4*							
rs1800759(G > T)	GG	77(0.688)	130(0.677)			1	1
	GT	33(0.295)	54(0.281)	0.906	1	1.032(0.615-1.730)	1.132(0.524-2.441)
	TT	2(0.017)	8(0.042)	0.270	0.810	0.422(0.087-2.039)	0.464(0.096-2.251)
HWE *p*-value		0.469	0.432				
Allele	G	187(0.835)	314(0.818)			1	–
	T	37(0.165)	70(0.182)	0.593		0.888(0.573-1.375)	–
*CLOCK*							
rs1801260(T > C)	AA	92(0.821)	167(0.870)			1	1
	AG	19(0.170)	23(0.120)	0.226	0.678	1.500(0.776-2.898)	1.471(0.766-2.823)
	GG	1(0.009)	2(0.010)	0.937	1	0.908(0.081-10.145)	1.151(0.533-3.487)
HWE *p*-value		0.986	0.245				
Allele	A	203(0.906)	357(0.930)			1	–
	G	21(0.094)	27(0.070)	0.301		1.368(0.754-2.482)	–

^a^: Adjusted for age, sex by logistic regression

*p*^corr^: *p* value is adjusted by Bonferroni correction

### Haplotype analysis of the *HCRTR2* SNPs

Haplotypes were constructed for cases and controls, and eight major haplotypes with frequency more than 3% were identified (Table 3). Among eight haplotypes, only H1-GTGGGG showed significant difference (44.7% vs. 53.1%, OR = 0.689, 95% CI =0.491~0.966, *p* = 0.030), while the other haplotypes (H2, H3, H4, H5, H6, H7 and H8) had no significant difference between the cases and controls (Table 3).

**Table 3:** Haplotype analysis of the *HCRTR*2 SNPs between cases and controls

Haplotypes ^a^	Case frequency	Control frequency	*p* -value	OR (95% CI)
H1-GTGGGG	100.11(0.447)	203.74(0.531)	0.030	0.689 [0.491~0.966]
H2-GTGGAG	26.31(0.117)	29.18(0.076)	0.094	1.602 [0.919~2.794]
H3-AGGAAG	25.17(0.112)	32.34(0.084)	0.270	1.284 [0.728~2.264]
H4-AGGGGG	14.91(0.067)	18.71(0.049)	0.369	1.378 [0.683~2.781]
H5-GGGAAG	8.55(0.038)	17.19(0.045)	0.678	0.838 [0.362~1.936]
H6-GTAGGA	11.05(0.049)	11.89(0.031)	0.262	1.608 [0.696~3.713]
H7-ATGGGG	6.39(0.029)	18.79(0.049)	0.213	0.564 [0.226~1.406]
H8-ATGGAG	8.06(0.036)	12.68(0.033)	0.866	1.081 [0.439~2.657]

^a^Haplotypes were omitted if the estimated haplotype probability was less than 3%. Order of polymorphisms: rs10498801-rs3122156-rs9357855-rs2653342-rs3800539-rs2653349.Global *X2* is 9.928, *df* = 8, *p* = 0.277

### Multiple logistic regression

Nine variants (rs10498801, rs2653342, rs2653349, rs3122156, rs3800539, rs9357855, rs1800759, rs1801260 and gender) were included in the multivariate analysis (stepwise backward LR), from which two variants (rs3800539 and rs1801260) were included in the final multiple regression analysis. As shown in Table 4, no significant difference was detected in the distributions of seven SNPs between patients and controls, after adjusting by multiple logistic regression analysis.

**Table 4:** Multiple logistic regression of the relation between the 8 polymorphisms and CH

SNP	Estimate	Wald	*p*-value	Point estimate OR (95% CI)
rs3800539 (GG Vs. GA + AA)	0.417	0.813	0. 063	1.513(0.654~3.912)
rs10499801 (GG Vs. GA + AA)	0.351	0.409	0.653	1.481(0.495~4.354)
rs3122156 (TT Vs. TG + GG)	0.076	0.038	0.824	1.125(0.554~2.186)
rs9357855 (GG Vs. GA + AA)	0.186	1.210	0.845	1.325 (0.248~3.712)
rs1800759 (GG Vs. GT + TT)	−0.015	0.018	0.913	0.986(0.635~1.531)
rs1801260 (CC Vs. CT + TT)	−0.682	0.432	0.362	1.482(0.539~2.984)
rs2653342 (GG Vs. GA + AA)	0.192	0.181	0.963	1. 231 (0.423~3.325)
rs2653349\ (GG Vs. GA + AA)	0.293	0.456	0.993	1.382(0.615~3.464)
Sex (male Vs. female)	0.187	0.154	0.868	1.236 (0.572~36.491)

## Discussion

As far as we are aware, the present study is the first to explore molecular evidence for association of different genotypes of SNPs in *HCRTR2*, *ADH4* and *CLOCK* genes with CH in Chinese Han population. The molecular genetic profiles of CH are mainly derived from the Western populations. Rainero et al. (2004) studied polymorphisms of the *HCRTR1* and *HCRTR2* genes, and revealed obvious difference for the polymorphisms of the *HCRTR2* rs2653349 loci between case and control groups. Compared with GA/AA genotype carriers, homozygous carriers of the *HCRTR2* 1246 GG genotype had the 5-fold increased CH risk (OR = 5.06, 95% CI = 1.99-13.64, *P* = 0.0002) [16]. Schurks et al. (2006) repeated the study with 226 cases of CH patients in Germany, conforming the 2-fold increased risk of carriers with homozygous G allele [17]. However, no statistically significant association of the polymorphism of *HCRTR*2 rs2653349 with CH was found in several case-control studies in Denmark, Sweden and the UK populations. One reason may be due to the small sample size in these studies, genetic background and environmental exposures [18]. Weller et al. (2015) genotyped a large sample of Dutch CH patients (575) and controls (874), and confirmed positive association of *HCRTR2* rs2653349 polymorphism with CH incidence by meta-analysis [15]. In the present study, no statistically significant correlation was found between *HCRTR2* rs2653349 and CH in a Chinese Han population consisting of 112 CH patients and 192 controls. Interestingly, we found that the frequency of the rs3800539 GA genotypes was significantly higher in cases than in controls (48.2% vs.37.0%). Moreover, the GA genotypes was detected to associate with a higher CH risk (OR = 1.483, 95% CI: 0.564-3.387, *p* = 0.038), however, no significant was found after Bonferroni multiple correction. Under the circumstances, the possible explanation is that small sample size could decrease statistical power. Furthermore, we would need a larger sample size to validate differences. Haplotype analysis of the *HCRTR2* SNPs revealed that only H1-GTGGGG had significant lower frequency in cases than in controls (44.7% vs. 53.1%, OR = 0.689, 95% CI =0.491~0.966, *p* = 0.030). The further study in the *HCRTR2* gene should focus on effects of haploid factors on CH.

*ADH4* gene, encoding an enzyme that plays an important role in the metabolism of alcohol, is an interesting candidate gene for CH. To date, there is little consistent evidence for the association of *ADH4* SNPs rs1126671 and rs1800759 with CH. Eising et al. (2017) revealed no association of *ADH4* with previously reported pathogenic mechanisms [30]. Previous studies reported contradictive results, which were dependent on variance in genotype, allele, and haplotype frequencies among the different populations. Rainero et al. (2010) found that the rs1126671 located in exon 7 of the *ADH4* gene was associated with an increased risk for CH in Italian case-control study, and the carriers with homozygous rs1126671 AA genotype had more than 2-fold CH risk than those with GG/GA genotypes (OR = 2.33, 95% CI = 1.25 4.37, *P* = 0.006) [23]. In addition, Zarrilli et al. (2015) also found an association between the *ADH4* SNP rs1800759 and CH [24]. However, the association was not supported by the case control study of Fourier et al. (2016), who reported no association of the *ADH4* SNPs rs1126671 and rs1800759 with CH using a largest Sweden population [25]. Similarly, the data of our study did not support an association of the *ADH4* SNPs rs1126671 and rs1800759 with CH. Interestingly, the present study confirmed that rs1126671 is not polymorphic in the Chinese Han population. This result is consistent with the results of PUBMED SNP library. The possible explanation for these results are as follows: Chinese patients have some different clinical profiles of CH from the Western ones, showing a relatively low prevalence of chronic CH, pain sites mainly focused on areas distributed by the first division of the trigeminal nerve, a low frequency of restlessness and absent aura. Therefore, *ADH4* rs1126671 could not be considered as a biomarker for screening CH in Chinese case-control group.

The present study did not provide supportive evidence for significant association of *CLOCK* gene rs1801260 with CH in Chinese Han population. A large part of these studies have proved this result, for instance, Rainero et al. (2005) reported that there was no correlation between the *CLOCK* gene 3092 T- > C (rs1801260) and CH in an Italian CH case-control sample (210 patients and 107 controls) [27], which is supported by subsequent studies of Zarrilli et al. (2015) and Cevoli et al. (2008) [24, 28]. Recently, Fourier et al. (2017) found a significant association of *CLOCK* gene rs12649507 with CH (*p* = 0.0069, OR = 1.29, 95% CI = 1.08~1.54) in a large Swedish CH case-control sample (449 patients and 677 controls) [29], strengthening the hypothesis of the involvement of circadian rhythm in CH. No significant association of *CLOCK* rs1801260 with CH was statistically detected in the present study, consistent with the previous results. Although a significant association with CH in Chinese case–control group was not found, *CLOCK* as a candidate gene for screening CH could not be excluded in the future study.

## Conclusion

In summary, this study is the first report to evaluate the association between CH and the *HCRTR2, ADH4* and *CLOCK* genes in Chinese Han population. The results suggest that the HCRTR2 (rs10498801, rs2653342,rs2653349, rs3122156, rs3800539, rs9357855), *ADH4*(rs1126671、rs1800759) and *CLOCK* (rs1801260) are not genetic risk factors for CH in the Chinese Han population. However, haplotype analysis found H1-GTGGGG was linked to a reduced CH risk.

## Additional files

Additional file 1: Table S1. The sequences of the primers in this study. (DOCX 12 kb)

## Abbreviations

*ADH*: Alcohol dehydrogenase
CH: Cluster headache
*CLOCK*: Circadian Locomotor Output Cycles Kaput
GPCRs: G-protein-coupled receptors
hypocretin, HCRTs: Hypothalamus gastrin-releasing peptides
SNPs: Single nucleotide polymorphisms

## References

[1] Dong Z, Di H, Dai W, Pan M, Li Z, Liang J, Zhang M, Zhou Z, Liu R, Yu S (2013). Clinical profile of cluster headaches in China - a clinic-based study. J Headache Pain.

[2] Headache Classification Committee of the International Headache Society(IHS) (2013). The international classification of headache disorders, 3rdedition (beta version). Cephalalgia.

[3] Schurks M (2010). Genetics of cluster headache. Curr Pain Headache Rep.

[4] Taga A, Russo M, Manzoni GC, Torelli P (2015). Familial cluster headache in an Italian case series. Neurol Sci.

[5] Russell MB, Andersson PG, Thomsen LL (1995). Familial occurrence of cluster headache. J Neurol Neurosurg Psychiatry.

[6] El Amrani M, Ducros A, Boulan P, Aidi S, Crassard I, Visy JM, Tournier-Lasserve E, Bousser MG (2002). Familial cluster headache: a series of 186 index patients. Headache.

[7] Leone M, Russell MB, Rigamonti A, Attanasio A, Grazzi L, D'Amico D, Usai S, Bussone G (2001). Increased familial risk of cluster headache. Neurology.

[8] Couturier EG, Hering R, Steiner TJ (1991). The first report of cluster headache in identical twins. Neurology.

[9] Roberge C, Bouchard JP, Simard D, Gagne R (1992). Cluster headache in twins. Neurology.

[10] Sjaastad O, Shen JM, Stovner LJ, Elsas T (1993). Cluster headache in identical twins. Headache.

[11] Russell MB, Andersson PG, Thomsen LL, Iselius L (1995). Cluster headache is an autosomal dominantly inherited disorder in some families: a complex segregation analysis. J Med Genet.

[12] Leone M, Proietti Cecchini A (2017). Advances in the understanding of cluster headache. Expert Rev Neurother.

[13] Li SB, Jones JR, de Lecea L (2016). Hypocretins, neural systems, physiology, and psychiatric disorders. Curr Psychiatry Rep.

[14] Nixon JP, Mavanji V, Butterick TA, Billington CJ, Kotz CM, Teske JA (2015). Sleep disorders, obesity, and aging: the role of orexin. Ageing Res Rev.

[15] Weller CM, Wilbrink LA, Houwing-Duistermaat JJ, Koelewijn SC, Vijfhuizen LS, Haan J, Ferrari MD, Terwindt GM, van den Maagdenberg AM, de Vries B (2015). Cluster headache and the hypocretin receptor 2 reconsidered: a genetic association study and meta-analysis. Cephalalgia.

[16] Rainero I, Gallone S, Valfre W, Ferrero M, Angilella G, Rivoiro C, Rubino E, De Martino P, Savi L, Ferrone M, Pinessi L (2004). A polymorphism of the hypocretin receptor 2 gene is associated with cluster headache. Neurology.

[17] Schurks M, Kurth T, Geissler I, Tessmann G, Diener HC, Rosskopf D (2006). Cluster headache is associated with the G1246A polymorphism in the hypocretin receptor 2 gene. Neurology.

[18] Baumber L, Sjostrand C, Leone M, Harty H, Bussone G, Hillert J, Trembath RC, Russell MB (2006). A genome-wide scan and HCRTR2 candidate gene analysis in a European cluster headache cohort. Neurology.

[19] Rozen TD, Fishman RS (2012). Cluster headache in the United States of America: demographics, clinical characteristics, triggers, suicidality, and personal burden. Headache.

[20] Edenberg HJ (2000). Regulation of the mammalian alcohol dehydrogenase genes. Prog Nucleic Acid Res Mol Biol.

[21] Buervenich S, Sydow O, Carmine A, Zhang Z, Anvret M, Olson L (2000). Alcohol dehydrogenase alleles in Parkinson's disease. Mov Disord.

[22] Luo X, Kranzler HR, Zuo L, Yang BZ, Lappalainen J, Gelernter J (2005). ADH4 gene variation is associated with alcohol and drug dependence: results from family controlled and population-structured association studies. Pharmacogenet Genomics.

[23] Rainero I, Rubino E, Gallone S, Fenoglio P, Negro E, De Martino P, Savi L, Pinessi L (2010). Cluster headache is associated with the alcohol dehydrogenase 4 (ADH4) gene. Headache.

[24] Zarrilli F, Tomaiuolo R, Ceglia C, Lombardo B, Izzo B, Castaldo G, Pastore L, De Simone R (2015). Molecular analysis of cluster headache. Clin J Pain.

[25] Fourier C, Ran C, Steinberg A, Sjostrand C, Waldenlind E, Carmine Belin A (2016). Screening of two ADH4 variations in a Swedish cluster headache case-control material. Headache.

[26] Ofte HK, Tronvik E, Alstadhaug KB (2015). Lack of association between cluster headache and PER3 clock gene polymorphism. J Headache Pain.

[27] Rainero I, Rivoiro C, Gallone S, Valfre W, Ferrero M, Angilella G, Rubino E, De Martino P, Savi L, Lo Giudice R, Pinessi L (2005). Lack of association between the 3092 T-->C clock gene polymorphism and cluster headache. Cephalalgia.

[28] Cevoli S, Mochi M, Pierangeli G, Zanigni S, Grimaldi D, Bonavina G, Torelli P, Manzoni GC, Cortelli P, Montagna P (2008). Investigation of the T3111C CLOCK gene polymorphism in cluster headache. J Neurol.

[29] Fourier C, Ran C, Zinnegger M, Johansson AS, Sjöstrand C, Waldenlind E, Steinberg A, Belin AC (2017) A genetic CLOCK variant associated with cluster headache causing increased mRNA levels. Cephalalgia. doi: 10.1177/0333102417698709. [Epub ahead of print] 10.1177/0333102417698709 28466652

[30] Eising E, Pelzer N, Vijfhuizen LS, Vries B, Ferrari MD, Hoen PA, Terwindt GM, van den Maagdenberg AM (2017). Identifying a gene expression signature of cluster headache in blood. Sci Rep.

