# Supplementary material for: Genetic association of HCRTR2, ADH4 and CLOCK genes with cluster headache: a Chinese population-based case-control study
Source: J Headache Pain. 2018 Jan 9;19(1):1. doi: 10.1186/s10194-017-0831-1 (PMC5760492; doi:10.1186/s10194-017-0831-1)
Supplement: Supplementary file 1 — The sequences of the primers in this study. (DOCX 12 kb) [file 10194_2017_831_MOESM1_ESM.docx]

Additional file 1: Table S1 The sequences of the primers in this study

| SNP_ID | Forward PCR primers | Reverse forward PCR primer | Extended primer sequence |
| --- | --- | --- | --- |
| rs3122156 | ACGTTGGATGGCTCACACAGTTATGGAGAC | ACGTTGGATGCTCTTTTAGCATGTGATGCC | GACCAAGGTCAAGGG |
| rs1801260 | ACGTTGGATGCCTGGAATTAGTTGGCAGAG | ACGTTGGATGCAGGCACCTAAAACACTGTC | GAGGTGATCATAGGGGCA |
| rs1126671 | ACGTTGGATGTGGACTGTACAACCGCAGG | ACGTTGGATGTACGGCCGATTATTAGCTCC | TCGGGTAGCAAAGGATTGACT |
| rs1800759 | ACGTTGGATGGGGCTGTGAATTACAGCAAC | ACGTTGGATGCCGTGTCCTATAATGAGCTG | GGGGCAACAAAGGAGAAAAGG |
| rs2653342 | ACGTTGGATGAAGATAGTCTCGCTGTCATC | ACGTTGGATGCCCAGCACTTTCTCCTATTC | TTTGTATCCCTATAAATAGCAC |
| rs2653349 | ACGTTGGATGCACATTGAGGATGCTAATTGG | ACGTTGGATGATAAAGCAGATCCGAGCCAG | TTGGCAAATACCAAAAGCACAA |
| rs9357855 | ACGTTGGATGCCTCAATACCATTGCTAACC | ACGTTGGATGTCTCTAGCCCAGAAGCTATG | AATAGAACTATGGTTGTATAGATT |
| rs3800539 | ACGTTGGATGCCCAACCAGTGGGTTAAAAG | ACGTTGGATGCAAAGACCTGCCCTTGAATG | TAGTGAGAAGAAAAATAATTTATCC |
| rs10498801 | ACGTTGGATGCAGCCATCAAGTAGCATTTC | ACGTTGGATGCTGAGATTTGGAAAGAAGAG | AATATTTATTTTATTAAGATCACCAAAG |
